# Supplementary material for: Are carbon emissions trading and green financial instruments synergistic? -Comprehensive quantitative research based on content analysis
Source: PLoS One. 2024 Mar 7;19(3):e0298601. doi: 10.1371/journal.pone.0298601 (PMC10919601; doi:10.1371/journal.pone.0298601)
Supplement: S1 Data — (ZIP) [file pone.0298601.s001.zip › Data/地级市Z_F工作报告.zip!/╝¬┴╓/╦╔╘¡╩╨/└┤╫╘╣1⁄2╓┌║┼í╛┬φ┐╦╩2╛▌═°í┐_2019.docx]

各位代表：

现在，我代表市政府，向大会报告工作，请予审议，并请市政协各位委员提出意见。

一、2018年工作回顾

2018年是松原发展史上极不平凡的一年。习近平总书记亲临视察，为松原振兴崛起指明了方向、提供了遵循、注入了强大动力。在这一年里，面对宏观经济环境趋紧、经济下行压力加大、融资渠道收窄、自然灾害频发等诸多考验和挑战，在省委、省政府和市委的坚强领导下，在市人大、市政协的监督支持下，我们团结带领全市各族人民，抢抓机遇、乘势而上，砥砺拼搏、真抓实干，全面完成各项目标任务，多项工作发生显著变化，取得了来之不易的发展成绩。

——经济发展企稳向好。预计（下同）地区生产总值同比（下同）增长4%，地方级财政收入增长1.6%，均为3年来最好水平。固定资产投资增长5%，成功扭转下降态势。社会消费品零售总额增长5.5%，与上年持平。城乡常住居民人均可支配收入分别增长6%，均跑赢GDP增速。

——项目建设成果喜人。积极组织参加各类经贸活动100多场（次），引进3000万元以上项目104个，到位资金192亿元。扎实开展项目建设“三抓”“三早”行动和大联检活动，开复工5000万元以上项目127个，完成投资191亿元，分别增长13.4%和7.7%，项目支撑作用日益凸显。

——城乡面貌焕然一新。实施基础设施项目74个，完成投资41亿元，一批关乎城市运行、商家经营和百姓生活的市政工程全面完工。大力开展市容环境综合整治，中心城区净化绿化美化亮化实现提档升级。全力实施农村人居环境综合整治，乡容村貌发生了历史性变化。

——“三大攻坚”战果显著。防范化解金融风险攻坚战全面打响，圆满完成财政刚性支出任务，按期偿还平台等债务51.8亿元，有效化解银行业金融机构风险，重拳整治民间借贷乱象，守住了不发生系统性区域性金融风险的底线。污染防治攻坚战提速发力，高效整改“绿盾行动”、中央环保督察等反馈问题，生态环境明显改善。精准脱贫攻坚战再获佳绩，45个村、15251名贫困群众如期脱贫，贫困发生率降低近1个百分点。

——社会治理不断加强。狠抓信访大排查、大化解，一大批历史积案得到有效解决。扎实开展“安全生产治理年”活动，工矿商贸和生产经营性交通事故分别下降52.2%和51.5%。深入开展扫黑除恶、“三打击一整治”等行动，群众的安全感和满意度进一步提升。

一年来，主要做了以下工作：

（一）抓调整、促转型，产业升级迈出新步伐。工业经济企稳向好。总量稳步扩大。持续稳油增气，完成油气产量当量473.5万吨，油气开采业增加值增长2%。全力支持重点企业上产扩能，石油机械化学助剂等项目竣工投产，嘉吉生化200万吨玉米深加工等项目开工建设，骨干企业进一步壮大。积极促进续建项目投产达效，鲁花10万吨花生油生产等一批项目建成投产。规上工业增加值增长5%。增量扎实培育。全力推动新签约项目落地建设，天成实业10万吨废矿物油综合利用、鹏鸿智能家居等项目实现当年开工、当年投产，投资百亿元的山鹰100万吨环保造纸项目落户松原。成功向上争取燃料乙醇生产指标30万吨。存量有效优化。着力激活僵尸企业和闲置产能，通过帮扶、嫁接、重组等方式，盘活“双停”企业73户，激活存量资产23亿元。充分发挥助保金池和产业引导资金作用，扎实开展银企保对接，设立工业和信息化专项奖励资金2400万元，新增规上工业企业31户。质量持续提升。加快推动企业改造升级，长山热电厂耦合发电、长山化肥厂15万吨复合肥、新创生化18万立方米纤维板等项目投入生产，新研牧神农机改扩建项目全面完工，百年汉克恒康制药项目完成主体工程。众诚油页岩示范基地获批国家原位开采工程示范分中心，双井抽油机研发被列为省级技术创新工程，和元生物等3家公司被认定为高新技术企业，三马电子等19家公司获评科技型中小企业。

现代农业提质增效。一产增加值增长2.5%。战胜严重旱灾，粮食总产量达到140亿斤，跃居全省第2位。围绕设施建设强农业。松原灌区骨干工程进入收尾阶段，河湖连通工程顺利推进，新增高标准农田、高效节水灌溉农田43万亩。围绕绿色有机强农业。松原国家农业科技园区顺利通过科技部复评。锦虹农业科技产业园一期等重点项目建成投产。投资5500万元，新建绿色农业示范基地67个。“三品”作物种植面积突破320万亩，认证数量达到316个。炭泉黑豆、乾安羊肉被列入国家地理标志产品保护名录。前郭县获批国家农产品质量安全县。成功举办中国·乾安杂粮产业峰会、中国·扶余花生暨杂粮杂豆产业博览会，“黄金小米”荣获中国绿色食品博览会金奖。围绕集约增效强农业。调减籽粒玉米种植面积45万亩，新增大豆种植面积70万亩、棚菜和中药材等特色作物种植面积20万亩。增加新型农业经营主体1361个；流转土地416万亩，占家庭承包耕地面积33%。乾安县入选全国率先基本实现全程机械化示范县。松粮集团跻身中国大米加工企业50强。围绕精品养殖强农业。新建标准化规模养殖场（小区）80个。中粮200万头生猪产业化等项目实现扩能升级。乾华美利奴羊培育项目填补国内多项空白。

服务业发展势头良好。服务业增加值增长5.5%。旅游业发展态势迅猛。《查干湖生态旅游发展规划》完成编制。查干湖景区服务功能进一步完善，野鸭湾等特色景点广受游客“点赞”。威尼斯水上乐园一期等项目进入内装阶段。接待游客数量和旅游综合收入分别增长11.7%和16.9%。商贸物流业发展向好。华隆农贸服务中心、迪卡侬运动商场等项目建成运营。松原农副产品展示展销中心在舟山设立。我市被列为全省唯一城乡高效配送试点市，瑞禾仓储物流园入驻企业达117户，豪森冷链仓储物流配送项目投入运营。快递业经营收入增长24%。房地产业发展持续回升。全面整顿和规范房地产市场，土地收储和净地拍卖供应扎实启动。完成投资40亿元，开发商品房341万平方米。销售商品房6718套、二手房1.3万套，分别增长37%和13.7%。金融业发展步伐加快。银行业金融机构存贷款余额按可比口径分别增长8%和6.3%。“三押”融资额突破288亿元。40户企业在省股权交易所挂牌。农村金融服务站实现乡镇全覆盖。新型服务业态发展提速。查干湖机场被列为全国通航业务收费试点，成功引进4家航空企业。两江高科技孵化产业园和吉油众创空间稳健运营，孵化科技企业超过50户。

（二）抓改革、破藩篱，发展活力实现新释放。关键领域改革不断深化。政府机构改革全面启动，有序开展。“放管服”改革深入推进，向经开区下放权力529项。行政职权全面调整，公布权力清单3765项。国企改革稳步推进，吉林油田公司“三供一业”完成分离移交。农业农村改革扎实推进，土地承包经营权确权登记颁证、集体产权制度改革清产核资基本完成，农垦改革完成垦区集团化、农场企业化改造，国有林场改革顺利通过省级验收。此外，财政国库集中支付、公立医院综合改革、文化市场综合执法改革成效显著。营商环境持续改善。硬环境不断强化，水电气路等配套设施进一步完善，重点园区实现“五通一平”；软环境持续优化，在全省率先确定四批1197项“只跑一次”事项，开创了“码上查、马上办”等政务服务新模式，实现市本级公共资源交易流程全程电子化。松原海关获批设立。民营企业户数增长27.6%；民营经济主营业务收入达到2438亿元，增长5%。

（三）抓建管、强功能，城乡发展呈现新气象。市中心城区建设扎实推进。人防工程、景观照明、农贸市场、停车场等专项规划完成编制。建设街、扶余大路等5条道路改造全面完工。团结街、成吉思汗大街等5条地下综合管廊投入使用。海绵城市一期工程有序推进，无名泄支综合整治实现水体不黑不臭。城区4个出入口综合改造顺利完成，松原东、查干湖收费站启动拓宽改造。松原大路、建华路等街路完成绿化彩化升级，“拆墙透绿”工程启动实施，新增绿地12.7万平方米。松原火车站等33幢标志性建筑和主要街路实现高品位亮化。县乡村建设取得明显成效。扶余市棚户区改造、前郭县街路建设、长岭县亮化升级、乾安县绿化美化等工程顺利竣工，县城面貌进一步改观。陶赖昭、长山、太平川、让字等重点镇基础设施不断完善。新建农村公路814公里，改造农村厕所30580个，绿化美化村屯208个，打造美丽乡村7个、美丽庭院2000个、干净人家5000户。城市管理力度不断加大。“数字松原”地理空间框架项目投入运营。市城市管理行政执法局组建成立。车辆乱停、市场外溢、城市野广告和焚烧冥纸等问题得到全面整治，静动态交通治理不断强化，群众生活环境更加规范有序。立体化交通格局日趋完善。松原火车站站前广场升级改造全面完成。查干湖机场功能完善项目有序推进，旅客吞吐量突破16万人次。五右、京哈、双嫩高速松原段开工建设。生态环境持续向好。累计投入资金7.8亿元，中央和省环保督察反馈问题分别办结98.5%和88.8%，查处环境违法案件213件。打响蓝天保卫战。淘汰和改造燃煤锅炉245台，淘汰黄标车2680辆，取缔露天烧烤摊点2800多处，实现秸秆禁烧和综合利用齐头并进。全年空气优良天数达到325天，增加10.6%。打响碧水保卫战。全面落实河长制、湖长制，《查干湖生态治理保护规划》完成编制，清理河湖污泥和垃圾10万立方米，治理污水口8处，提标改造污水处理厂5座，增设水质检测站（点）17个，“两江一河”4个国控断面水质全面达标。首部地方性法规——《松原市饮用水水源保护条例》颁布实施，拆除水源地违章建筑9处。打响黑土地保卫战。拆除粘土砖窑77个，关停和搬迁养殖场72个，处理陈腐垃圾221万吨、垃圾渗滤液3.2万立方米，市县建成区生活垃圾实现100%无害化处理，城乡垃圾收转运体系初步建成，土壤面源污染防治工作有效开展。植树造林23.5万亩，还林还草2.3万亩。前郭县获评国家级“绿水青山就是金山银山”实践创新基地。

（四）抓民生、促和谐，人民生活获得新改善。地震灾区建成崭新家园。抗震救灾取得全面胜利，投入资金1.48亿元，妥善安置群众8000余人，农村受损房屋重建、修缮和加固全面完成，6571户受灾群众全部入住安全暖心房子。保障和改善民生深入推进。市县两级民生行动计划顺利实施，民生实事全部兑现承诺。新增城镇就业4.3万人次，转移农村剩余劳动力61万人次。2.3万名残疾人获得基本康复服务。退休人员养老金、失业保险金、城乡低保金和机关事业单位职工工资等全面提标。“情暖松原”等救助活动扎实开展。市社会福利院医养结合项目进入内装阶段。市救灾指挥中心建成使用。整治老旧小区40万平方米，改造棚户区住房3000套，发放住房公积金贷款8亿元，完成1.8万套无籍房产权登记颁证。社会事业全面进步。教育事业健康发展，良师工程深入实施，圆满实现地震期间平安高考，有效解决33所学校大班额问题，全面完成100所农村中小学供暖改造，宁江区九年一贯制学校、油区松江小学新教学楼投入使用。医疗事业稳步发展，“健康松原”活动深入推进，市中心医院异地新建项目和市食品药品检验中心全面建成，医疗影像云平台上线运营。文体事业蓬勃发展，市满族新城戏传承保护中心获评省级非遗传承基地，歌舞剧《天琴·神骏》获全省少数民族文艺会演特殊贡献奖，新建村级文化小广场31个。市体育馆投入使用，市冰上运动中心全面竣工，获省运会金牌数量创历史新高。此外，地面数字电视无线覆盖工程投入运营，统计工作服务经济发展能力显著增强，市政府网站入选中国“互联网+政务”优秀实践案例50强。社会治理进一步加强。“七五”普法深入开展。民族和宗教事务管理日益规范。以“事要解决”为根本，344件国省交办信访案件全部化解，1354件信访积案办结90%。强力开展安全生产“十大专项治理”，整改隐患9700多处。圆满完成“1828”警卫、查干湖冬捕节等重要安保维稳任务，扎实开展“八大”专项战役，打掉涉黑涉恶团伙73个，破获双辉肥业诈骗等案件2173起。

（五）抓管理、提效能，政府机关展现新形象。着力加强依法行政。自觉接受市人大及其常委会法律监督、工作监督和市政协民主监督。认真办理市人大代表建议、政协提案261件，办复率达100%，满意率达95%以上。不断强化审计监督和正风肃纪，办公用房、公务用车管理和“三公”经费支出全面规范。着力加强自身建设。扎实开展解放思想主题教育实践、干部作风大整顿、推动高质量发展大讨论、“五走进”等活动，大力推行清单上墙、挂图作战工作机制，全面落实“一岗五责”。坚持迎着问题上，切实强化责任担当、主动作为，各项决策部署高效落实，圆满完成国省两级巡视、督查等迎检任务110多次。

各位代表！过去一年，面对多年累积矛盾集中凸显、各类风险交织叠加的严峻形势，我们顶住压力、迎难而上，稳中求进、进中有为，取得了一系列重要突破，开创了振兴发展新局面！这些成绩的取得，是省委、省政府和市委统揽全局、坚强领导的结果，是市人大、市政协有力监督、鼎力支持的结果，是各地各部门和全市广大干部群众团结奋进、攻坚克难的结果。我代表市政府，向全市各族人民，向人大代表、政协委员，向各民主党派、工商联、人民团体和社会各界人士，向驻松部队、武警官兵、公安干警和中省直单位，表示崇高的敬意和衷心的感谢！

在看到成绩的同时，我们也清醒地认识到工作中存在的问题和不足，主要表现为“六不”：结构“不优”，“一油独大”的产业格局尚未根本改变，地方工业体量依旧偏小；新的经济增长点“不多”，新旧动能转换较为艰难，接续产业发展迟缓，经济增长后劲不足；“抓投资、上项目”思想树得“不牢”，高质量谋划项目、优先发展产业项目意识有待提高，重大优质项目、“航母类”项目数量较少，在广招商、招大商上还需高压紧逼、持续用力；新增财力“不足”，刚性支出不断加大，收支矛盾十分突出；资源优势转化“不快”，“原字号”产品深加工率低、附加值低，资源优势远未转化成发展优势；担当作为“不够”，一些部门和工作人员大局意识和责任意识不强，少数干部不想为、不敢为、不会为的现象依然存在。同时，我们的工作还面临四个“压力加大”的严峻挑战：宏观经济环境进一步趋紧，转型发展压力加大；部分群众生活仍然较为困难，特别是剩余贫困人口多为贫中之贫，改善民生和脱贫攻坚压力加大；生态修复和环境保护形势严峻，污染防治压力加大；城乡基础设施和人居环境欠账较多，集中补齐短板压力加大。对此，我们高度重视，将在今后工作中认真加以解决。

二、2019年主要工作

2019年是决胜全面小康、决战精准脱贫和污染防治攻坚之年，也是松原生态振兴、转型崛起的关键之年。做好今年的工作，意义尤为重大。我们要保持战略定力、增强发展信心、抢抓发展机遇。国家层面，重点抢抓习近平总书记视察松原、新一轮东北振兴、哈长城市群建设、长吉松产业转型升级示范区建设、东北西部生态经济带规划建设、长吉图规划纲要中期修规“六大机遇”。省级层面，重点抢抓东中西“三大板块”建设、“一主六双”区域战略布局、“数字吉林”建设“三大机遇”。千方百计赢得先机，积极争取各类支持，加速培育经济发展内生动力。

今年政府工作的总体思路是：深入贯彻习近平总书记对东北振兴和视察松原重要指示精神，全面落实中央省委市委全会、中央和全省经济工作会议决策部署，坚持稳中求进工作总基调，以推动高质量发展为主题，以供给侧结构性改革为主线，抢抓战略机遇，紧扣振兴崛起，抓住“一个统领”不动摇，建设“两个城市”不放松，打好“三大战役”不懈怠，锁定“六稳”目标，围绕“五个合作”，放大比较优势、深度开发资源、狠抓项目建设、培育壮大产业、补齐发展短板，统筹推进稳增长、促改革、调结构、惠民生、防风险各项工作，加快把“松原发展很有潜力”变成生动现实。

今年全市经济社会发展的主要预期目标是：地区生产总值增长5%左右，地方级财政收入保持正增长，全社会固定资产投资增长5%以上，社会消费品零售总额增长5%左右，单位GDP能耗下降3%左右，城乡常住居民人均可支配收入均增长6%左右，城镇登记失业率控制在4.5%以内。着力实施好“四大工程”：

（一）实施产业优化升级工程。促进工业提速增效。致力于打造东北新型工业基地，深度融入长春经济圈、环长春四辽吉松工业走廊，推动工业经济实现质与量“双提升”。打好转化牌。全力支持吉林油田公司和东北油气公司稳油增气，新增原油产能50万吨、天然气产能2.7亿立方米，必保油气产量当量突破470万吨。力促“原油”“原气”深度转化，谋划建设俄油、俄气加工转化项目，扎实推进油页岩原位转化先导试验示范区、长岭双坨子储气库建设。着力提高“原粮”等农产品就地加工转化率，推动盼盼食品二期、天源生化乙醇改造项目投入生产，开工建设爱普25万吨焦糖色素、九佳60万吨马铃薯加工项目，力推嘉吉生化100万吨大豆压榨项目落地建设。打好多元牌。致力于构建多点发力、多极支撑的工业格局，着力发展生物医药和生物质综合利用产业，支持百年汉克、和元生物等企业做大做强，开工建设山鹰100万吨环保造纸、禾木之家20万立方米秸秆纤维素板材项目；着力发展装备制造产业，依托新研牧神等骨干企业，积极发展农业机械、钻采配件、汽车零部件等装备制造，促进坤成电力1万吨镀锌电料生产等项目建成投产；着力发展新型肥料产业，推动源尚5万吨生物肥项目投产达效。打好升级牌。全力扶持工业企业由“低小散弱”向“高大精优”转型，扩大助保金池和产业引导资金规模，力推“小升规、规改股、股上市”，新增规上工业企业20户，争取30户企业完成股改、30户企业在省股权交易所挂牌。鼓励企业生产技术改造升级，落实研发投入财政补助等政策，推动松粮集团院士工作站获批设立，培育4个市级以上“双创”平台。

促进农业提质升级。着力完善现代农业“三大体系”，全力打造一批乡村振兴亮点工程，加快建设全国知名绿色农业城。向完善设施要产量。完成花敖泡蓄水调蓄主体工程和道字、有字等泡沼征收任务，加快推进河湖连通、引松入扶等工程建设，强力开展“大棚房”问题清理整治，新增高标准农田24万亩，确保正常年景粮食总产量突破150亿斤。向结构调整要品质。调减籽粒玉米种植面积20万亩，新增棚菜、中药材等特色作物种植面积30万亩。继续抓好海峡两岸生态农业合作先行实验区建设，新建绿色农业示范基地20个，增加“三品”作物种植面积10万亩。向集约经营要潜力。推动耕地加速流转，土地规模经营面积达到550万亩，占比提高6个百分点。着力培育市场经营主体，新增农民专业合作社、家庭农场1000个。向产业融合要活力。围绕打造一二三产业融合试验区，加快高油玉米、花生、马铃薯等原料种植基地建设，巩固扩大正邦等企业养殖规模，力推牧原100万头生猪产业化项目建成运营，促进农畜产品向“产加销”方向迈进。探索发展可视农业、文创农业、休闲农业、乡村旅游，着力培育产业融合新业态。向市场开发要效益。全面打响“善德良米”“查干湖大米”等品牌，扎实推进“我在松原有亩田”项目，进一步提升农产品知名度和竞争力。开展“互联网+高质量农产品”行动，强化与舟山市和京东商城、淘宝网战略合作，加快推进阿里巴巴未来农场建设，让更多农产品走向高端市场。

促进服务业提档升级。围绕打造哈长城市群服务业基地，加快发展生产性、生活性服务业，着力培育新的经济增长点。大力发展特色旅游业。高质量完成《查干湖生态保护与利用总体规划》修编，强力推动“四季查干湖”整体开发，启动查干湖生态特色小镇建设，争取查干湖景区晋升为5A级景区。以查干湖景区为引领，积极参与全省河湖草原湿地旅游大环线建设，深度开发湿地游、草原游、沿江游、冰雪游、民俗游等旅游产品，着力构建精品旅游线路，加快发展全域旅游。大力发展康养产业。着眼于打造吉林省西部康养中心，加快发展养生养老、温泉疗养、医养结合等产业，规划建设松花江沿岸康养带，积极推动一龄生命健康城、怡心园温泉养生等项目开工建设。大力发展商贸物流业。致力于打造东北地区特色农产品商贸物流中心，规划建设粮食、蔬菜、畜禽等区域交易市场，促进敬亿国际商贸物流城二期、居然之家商贸综合体、大润发超市江北店投入运营，开工建设新洋丰仓储物流项目。大力发展房地产业。规范管理房地产市场，开展清缴税费专项行动，全力推进征拆、收储、供地、拍卖“四统一”。完成投资37.5亿元，开发商品房350万平方米。大力发展金融业。探索开展订单、应收账款、库存商品“多权”抵质押。深入开展农村金融综合改革试点。扩大农村土地经营权、土地收益保证抵押贷款范围。提高农村金融服务站覆盖率。着力增强金融服务实体经济能力。大力发展数字经济。加快打造“数字松原”。加强移动通讯网络建设。积极发展大数据、电子商务等产业，推动华录集团扶余“数据湖”、盛达汽车电子商务大厦建成使用，加快浪潮集团云计算中心建设，力推科大讯飞孵化中心落户松原。

（二）实施城乡建管提质工程。以创建国家级卫生城和打造生态宜居城市为统领，着力建设功能完善、特色鲜明的美丽松原。高品位优化空间布局。突出地域、文化和民族特色，高质量修编《松原市城市总体规划》，完成铁南片区概念规划等编制工作，促进“多规合一”和市县乡村规划“多元合一”。扎实推进新设市辖区申报工作。高标准建设管理中心城区。全面加强路网建设，打通9条断头路，新建续建滨江大道、和平西路等24条道路，建设过街天桥3座，新建停车场10处。完成环保大街、和谐大街等5条地下综合管廊建设。加快海绵城市建设，改造城区涝点11个，铺设雨污分流管线22公里。完善供水供热设施，实施江北哈达山水库取水工程，新建改造供水管网59公里、供热管网29公里。着力提升城市品质，扎实推进圣湖补水工程，完成镜湖公园、扶余公园和行政广场改造，全面实施“拆墙透绿、见缝插绿”和江心岛绿化工程，深入推进主要街路亮化升级和楼体立面改造，大幅提升城市“颜值”和靓丽度。大力开展静动态交通专项整治、市容环境综合整治和集中拆违专项行动，启动架空线缆整治工程，严厉打击私搭乱建、乱停乱放、噪声扰民和黑车营运等行为，全面加强小区物业管理。高层次推进县城和镇村建设。以经营城市理念经营农村，推动各县（市）争创省级卫生城，着力完善县城功能、提升县城面貌。争取太平川镇晋升为全国重点镇和省级特色小镇。新建农村公路800公里。开展“农村人居环境整治重点年”活动，实现农村生活垃圾收转运全覆盖，改造农村厕所4.3万个，打造美丽乡村8个、美丽庭院2000个、干净人家5000户。高站位构建综合交通体系。围绕打造东北地区重要交通枢纽城市，积极参与中蒙俄经济走廊建设，增辟查干湖机场航线，完成机场功能完善项目，新建通航机坪2万平方米。力促松原至长春段动车时速提至200公里。推动五右高速松榆段竣工通车，完成京哈、双嫩高速松原段年度建设任务，开工建设长太高速。促进市客运枢纽工程和宁江客运站建成使用。

（三）实施“三大攻坚”突破工程。持续打好防范化解金融风险攻坚战。加强政府债务管理，规范举债行为，强化限额管理和预算约束，妥善处置政府存量债务，促进松发集团等平台实现转型、良性发展。加强金融监管，全力清收不良贷款，开展“清剿老赖”专项行动，规范担保公司、融资租赁公司发展运营，着力净化金融生态环境。加强民间借贷风险管控，严厉打击非法集资、金融诈骗等违法犯罪活动。

持续打好污染防治攻坚战。坚决完成中央和省环保督察反馈问题年度整改任务，打好打赢蓝天、碧水、黑土地、草原湿地“四大保卫战”。实施清洁空气行动，全面完成燃煤小锅炉淘汰改造，严控机动车尾气、建筑施工扬尘和挥发性有机物污染，强化餐饮行业油烟治理，加强秸秆露天焚烧管控，力争空气质量优良天数达到93%以上。实施清洁水体行动，强力推进查干湖水环境综合治理，投资46亿元，实施生态移民安置等项目7个，加快退耕轮耕、削减退水、还湿还林还草，确保查干湖生态治理初见成效。深入落实河长制，扎实开展河湖岸线管理保护和水体清洁整治。着力推进中心城区、县城和重点镇污水处理厂建设，实现无名泄支水体“长治久清”。加强水源地保护，确保群众饮用水绝对安全。实施清洁土壤行动，开展土地污染详查，构建土壤环境监测网络，强化农业面源污染防治和工业污染源头治理，加强养殖场环境卫生综合整治，扩大生活垃圾无害化处理覆盖面。深入开展“绿化松原大地”行动，大力实施“三北”防护林五期、林地清收还林等生态修复工程。

持续打好精准脱贫攻坚战。紧扣“两不愁、三保障”等要求，推动剩余16个贫困村、16643名贫困群众全部脱贫。产业带动促脱贫。加快发展瓜果、食用菌等特色种植，做大做强设施农业、庭院经济，大力发展乡村旅游、光伏发电和农村电商。完善设施促脱贫。加强贫困村路网建设，持续推进危房改造，加快电网改造升级，巩固提升饮水安全工程。统筹事业促脱贫。继续免除高中贫困学生学杂费用，开展扶贫巡回医疗体检等活动，扩大文化小广场覆盖面，加大贫困群众就业技能培训力度。强化保障促脱贫。推动无劳动能力等贫困群众纳入低保范围，健全农村残疾人关爱服务体系，大力开展民企帮扶“光彩行动”。

（四）实施民生福祉增进工程。扎实推进普惠性、基础性、兜底性民生建设，着力抓好市本级38个民生项目和县级民生行动计划，让人民群众拥有更多获得感。大力促进就业创业。新增城镇就业3.3万人次。转移农村劳动力52万人次。启动全省首个家庭服务业产业园试点建设。实施蓝领培训计划。发放创业担保贷款6000万元。织密织牢社会保障救助网。进一步提高基本养老金、失业保险金、新农合补助和低保对象大病救助等标准。发放社会医疗救助和临时救助资金3000万元。推动市江南救灾物资储备库建成使用。谋划建设国家级防震减灾中心。加快改善群众居住条件。强力实施棚户区改造三年行动计划，年内改造住房6112套以上。综合整治老旧小区20万平方米。改造39幢老旧楼地沟管网。完成4.29万套无籍房产权登记颁证。改造农村危房5000户。优先发展教育事业。科学调整城区教育规划布局。全面整合城乡教育资源。着力强化师资力量。强力开展在职教师有偿补课、兼职取酬整治行动，谋划建设教育培训服务集聚区。建成职业技术学院省级学前教育实训基地，推动市实验高中科技图书楼、油田供应小学教学楼、市第二幼儿园投入使用。不断提高健康服务水平。实施改善医疗服务行动计划。加强医联体建设管理。加快发展中医药事业。推动市中心医院、市儿童医院、宁江区同心医院投入使用，完成市妇幼保健院综合楼、市中心血站项目主体工程，启动市中医院异地搬迁，筹建市传染病防治医院。全力促进文体惠民。加强馆站院等公共文化服务阵地建设，深入实施文化精品展演工程，推动鼎润文化非遗传承体验中心投入使用。积极开展全民健身系列活动，开工建设市全民健身田径场。

为实施好“四大工程”，要着力强化“四大支撑”：

（一）突出项目引领，强化动力支撑。大力开展“项目建设年”活动，做优做强增量，增强发展后劲。深度谋划包装项目。把准国省政策导向、市场走向、企业动向，立足区位、交通、资源、产业等比较优势，精心谋划储备“四优”项目200个。广泛开展招商引资。围绕深化“五个合作”，积极开展以商招商、产业链招商和小分队招商，力争新引进重点项目100个、到位资金200亿元。强力推进项目建设。深入落实全省“三抓”“三早”行动部署，大力开展项目建设大联检、领导跟踪包保活动，全面提高项目建成率、投产率。全年开复工亿元以上项目126个，完成投资239亿元，分别增长7%和8%。

（二）突出激发动能，强化活力支撑。坚持以改革促发展，向创新要效益。着力深化体制机制改革。圆满完成政府机构改革，确保职责清晰、运转顺畅、效能提升。深入推进“放管服”改革，完善政务服务“一张网”，全力优化产业和基础设施项目审批线、服务群众办事线，确保3个工作日内可开办企业、50个工作日内可完成一般投资项目规划和施工许可审批。完成县乡财税体制改革，深入开展综合治税专项行动，积极达成耕地占补平衡指标交易。大力推进市县两级供水、供热等城市公共资源市场化运营。统筹推进事业单位、医药卫生、国有企业、农业农村等领域改革。着力优化营商环境。全面推动各类园区整合，加强基础设施建设和配套服务完善。推进“诚信松原”建设，开展优惠政策兑现专项行动，严厉查处涉软案件，加速打造东北地区投资洼地。着力激发民营经济活力。大力开展“民营企业服务年”活动，实施企业家培养和素质提升工程，加大人才引进力度，减免企业税费4亿元，扩大直购电市场交易范围。民营经济实现主营业务收入2560亿元、增加值595亿元，分别增长5%和3%。

（三）突出社会治理，强化安全支撑。持续提高治理法治化、智能化、专业化水平，为经济社会发展保驾护航。全力维护社会稳定。深入开展“大排查、大化解”、领导干部接访下访等活动，全面办结重点信访积案。抓好行业性、专业性人民调解中心建设。坚决维护民族团结。依法加强宗教管理。着力抓好安全生产。大力开展工矿商贸、道路交通、食品药品等领域专项治理，推动省级石化行业应急救援培训基地投入使用，加强震情监测研判，坚决遏制较大以上事故发生。有效维护社会治安。深入开展扫黑除恶、社会治安突出问题整治等专项行动，严厉打击电信诈骗等违法犯罪行为，坚决保障人民群众生命财产安全。

（四）突出政府建设，强化服务支撑。开展政府系统“作风建设提升年”活动，全面履行“一岗五责”，努力建设人民满意政府。坚持依法行政。自觉接受人大监督、政协监督和各方面监督，认真办理人大代表建议和政协提案。完善重大事项专家论证、合法性审查等决策程序，着力规范行政决策和执法行为。坚持务实为政。大兴真抓实干之风，树牢干工作“不在会场在现场”的鲜明导向。坚持不躲事、不怕事，对改革发展中遇到的矛盾敢于担当、主动回应。强化绩效考核和督查问责，承诺的事情必须兑现，确定的工作必须完成。坚持高效理政。完成市政务服务中心迁址改造，全面提升大厅综合服务能力。着力整合精简督查检查活动，大幅压缩会议和文件，让广大干部集中精力抓项目、跑招商、干实事。坚持从严治政。持之以恒落实中央“八项规定”，持续巩固作风大整顿活动成果，严控“三公”经费支出。切实加强党风廉政建设，严肃查处行政审批、工程建设和扶贫等领域腐败行为，坚决守护好政治生态的“绿水青山”。

各位代表！实干成就梦想，奋斗铸就辉煌！当前的松原，正处于爬坡过坎的艰难阶段、振兴崛起的关键时期。让我们在省委、省政府和市委的坚强领导下，抢抓机遇、迎难而上，真抓实干、开拓创新，为创造更加美好的明天努力奋斗，以优异的成绩向建国70周年献礼！
